# Crystal Structure of Carbonic Acid (H_2_CO_3_) at Elevated Pressures from Single‐Crystal Diffraction

**DOI:** 10.1002/chem.202501964

**Published:** 2025-07-09

**Authors:** Dominik Spahr, Elena Bykova, Lkhamsuren Bayarjargal, Maxim Bykov, Lukas Brüning, Valentin Kovalev, Victor Milman, Nico Giordano, Hanns‐Peter Liermann, Björn Winkler

**Affiliations:** ^1^ Goethe University Frankfurt Institute of Geosciences Altenhöferallee 1 60438 Frankfurt Germany; ^2^ Goethe University Frankfurt Institute of Inorganic and Analytical Chemistry Max‐von‐Laue‐Straße 7 60438 Frankfurt Germany; ^3^ Dassault Systèmes BIOVIA 22 Cambridge Science Park Cambridge CB4 0FJ United Kingdom; ^4^ Deutsches Elektronen‐Synchrotron DESY Notkestrasse 85 22607 Hamburg Germany

**Keywords:** carbonic acid, H_2_CO_3_, high‐pressure synthesis, raman spectroscopy, single crystal diffraction

## Abstract

Single crystals of carbonic acid (H_2_CO_3_) were synthesized in a laser‐heated diamond anvil cell at moderate pressures between 5 and 13 GPa by reacting H_2_O with CO_2_. Its monoclinic crystal structure (*P*2_1_/*n* with *Z* = 4) has been obtained from synchrotron single‐crystal X‐ray diffraction experiments at ≈8 GPa. The positions of the hydrogen atoms have been determined from the experimental data. Density functional theory‐based calculations in combination with experimental Raman spectroscopy confirmed the structural model derived from the diffraction data. This is the first single‐crystal structure solution of water‐free carbonic acid, H_2_CO_3_. The structural model provided here differs from structural models reported earlier for lower pressures derived from neutron powder diffraction data.

## Graphical abstract

Carbonic acid (H_2_CO_3_) was synthesized by a reaction of H_2_O with CO_2_ at moderate pressures and temperatures. For the first time, the crystal structure of an H_2_CO_3_ polymorph was determined by single‐crystal X‐ray diffraction. The structural model was confirmed by DFT‐based calculations in combination with experimental Raman spectroscopy.

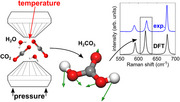

## 1 Introduction

The crystal structure of solid carbonic acid, H_2_CO_3_, and its high‐pressure behavior have long been disputed.^[^ ^1^, ^2^ ^]^ In fact, a determination of the crystal structure based on single‐crystal diffraction has not been published to date. The absence of a reliable crystal structure has prevented lattice dynamical calculations, which are needed for an unambiguous assignment of the vibrational spectrum of solid H_2_CO_3_, which, in turn, is required for remote sensing to identify the presence of solid carbonic acid in meteorites or on icy moons or planets.^[^ ^3^, ^4^, ^5^, ^6^, ^7^ ^]^ Furthermore, carbonic acid molecules may be building blocks in hydrous inorganic pyrocarbonates, such as Ba[H_4_C_4_O_10_][H_3_C_4_O_10_][H_2_CO_3_][HCO_3_].^[^ ^8^ ^]^ In order to be able to compute formation enthalpies of such compounds, the ground state structure of solid H_2_CO_3_ has to be known.

H_2_CO_3_ forms in small quantities by the reaction between H_2_O and CO_2_ in aqueous solution, but it dissociates rapidly again.^[^ ^9^, ^10^ ^]^ Two crystalline polymorphs (α‐ and β‐H_2_CO_3_) were proposed to exist, based on spectroscopic evidence.^[^ ^11^, ^12^, ^13^ ^]^ Later, α‐H_2_CO_3_ was revised to be CH_3_OCO_2_H (monomethyl ester of H_2_CO_3_), while β‐H_2_CO_3_ was assumed to be the only occurring polymorph, but its exact crystal structure remained elusive.^[^ ^14^ ^]^ In other experiments, solid H_2_CO_3_ was obtained by irradiation with particles or light of ice:CO_2_ mixtures.^[^ ^15^, ^16^ ^]^ However, in these experiments, the presence of solid H_2_CO_3_ was only inferred from spectroscopic studies.

An alternative route to obtain samples relies on high‐pressure experiments. A crystal structure of H_2_CO_3_‐*Pnma* was predicted by density functional theory (DFT) calculations at pressures >1 GPa. It was proposed that the computed vibrational spectra were in agreement with the experimentally obtained IR and Raman spectra measured at ≈4 GPa in a study reported earlier.^[^ ^17^, ^18^ ^]^ A combined neutron powder diffraction and DFT‐based study provided the first experimental crystal structure description on a deuterated sample at ≈2 GPa.^[^ ^19^ ^]^ The structure proposed in that study is monoclinic and H_2_CO_3_‐*P*2_1_/*c* substantially differs from H_2_CO_3_‐*Pnma*. In order to be able to refine the crystal structure in a phase mixture, where the diffraction signal was strongly dominated by CO_2_‐I, numerous restraints and constraints were introduced. The conformation of carbonic acid molecules in crystalline solids is therefore currently still unknown, given that the energy difference for *cis–cis* and *cis–trans* confirmations is small (≈1.6 kJ/mol).^[^ ^14^ ^]^ A related phase, carbonic acid monohydrate H_2_CO_3_.H_2_O, has been studied by single‐crystal diffraction at 6.5 GPa and can serve as a reference for the geometry of the H_2_CO_3_ molecule.^[^ ^20^ ^]^

In recent studies, we have shown that chemically simple new “conventional” carbonates, i.e., those containing nearly planar trigonally coordinated carbon atoms, such as Al_2_(CO_3_)_3_ or Be(CO_3_), can be synthesized from the corresponding oxide and CO_2_ in a laser‐heated diamond anvil cell (LH‐DAC) at moderate pressures (≈20 GPa) and elevated temperatures.^[^ ^21^, ^22^ ^]^ In these studies, the CO_2_ was loaded cryogenically as dry‐ice into the DAC, and after the synthesis of the new phases, their crystal structures were determined by synchrotron‐based X‐ray single‐crystal diffraction. This is an extension of the approach pioneered earlier for the synthesis of 3*d*‐transition metal carbonates.^[^ ^23^, ^24^ ^]^ We adapted this experimental approach using H_2_O and CO_2_ (see Supporting Information). The experiments in this study were carried out in an LH‐DAC at different pressure points between 3 and 15 GPa at elevated temperatures.

## 2 Results and Discussion

The phase diagram of CO_2_ shows several polymorphs depending on the *p*, *T* conditions.^[^ ^25^ ^]^ At pressures below ≈12 GPa, CO_2_‐I ($Pa\bar{3}$) is the stable phase up to its melting temperature.^[^ ^25^, ^26^ ^]^ This is in contrast to the phase diagram of H_2_O, where at low pressures several H_2_O‐ice phases have been reported.^[^ ^27^ ^]^ The stable phase in the pressure range investigated here is H_2_O‐VII ($Pn\bar{3}m$).^[^ ^27^, ^28^ ^]^

**Figure 1:**
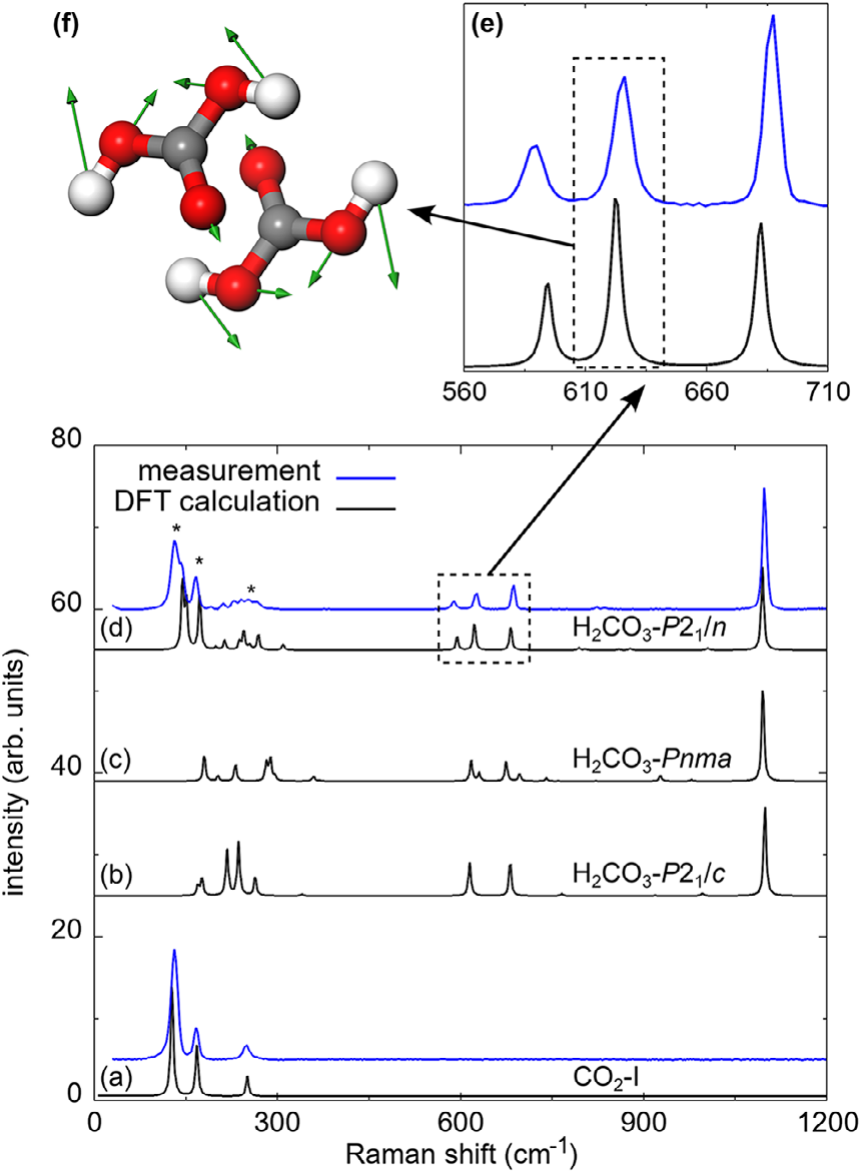
Raman spectroscopy at 9 GPa: a) Raman spectra for CO_2_‐I. b) Raman spectrum for hypothetical H_2_CO_3_‐*P*2_1_/*c*.^[^ ^19^ ^]^ c) Raman spectrum for hypothetical H_2_CO_3_‐*Pnma*.^[^ ^17^ ^]^ d) Raman spectra of H_2_CO_3_‐*P*2_1_/*n* after the synthesis. Peaks of CO_2_‐I are marked by an asterisk (*). e) Enlargement of the region between 560 and 710 cm^−1^. Experimental Raman spectra are shown in blue and DFT‐based calculations (rescaled by 1%–3 %) are shown in black. f) Eigenvector of the atomic displacements in the H_2_CO_3_ molecule for the characteristic Raman mode at ≈620 cm^−1^.

**Figure 2:**
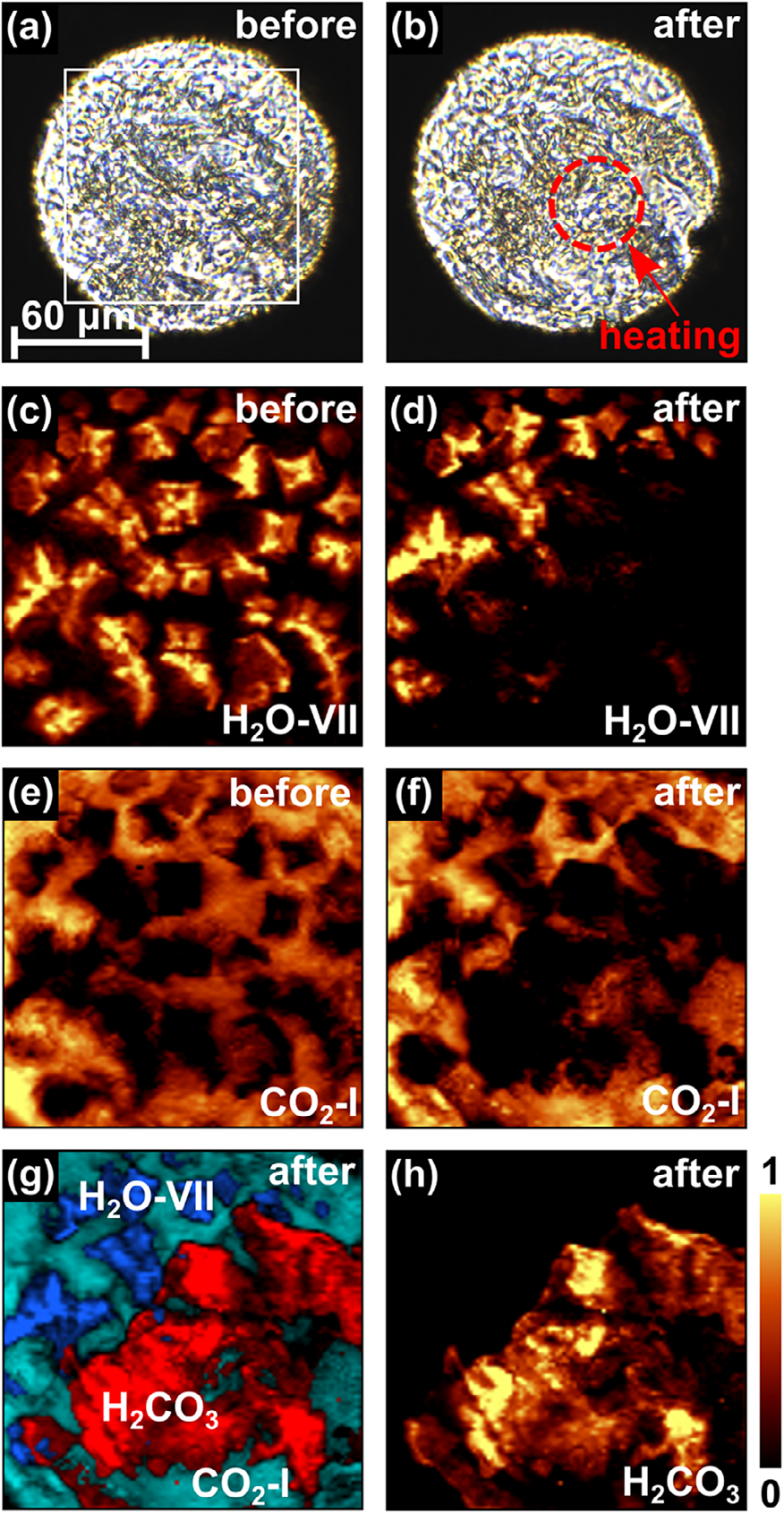
a,b) Sample chamber of the DAC with the H_2_O + CO_2_ mixture before and after laser heating at 10(1) GPa. 2D‐Raman maps of the distribution of c,d) H_2_O‐VII and e,f) CO_2_‐I before and after laser heating. g) Combined Raman maps of the phases present in the gasket hole after heating H_2_O‐VII (≈3100 cm^−1^), CO_2_‐I (≈130 cm^−1^), and H_2_CO_3_‐*P*2_1_/*n* (≈1195 cm^−1^). h) Raman map of H_2_CO_3_‐*P*2_1_/*n*.

In a Raman spectrum obtained at 9(1) GPa before the laser heating, we could identify the strong characteristic Raman modes of CO_2_‐I at low wavenumbers. The experimentally obtained Raman spectrum is in very good agreement with the Raman spectrum from our DFT‐based calculations (Figure 1a). In addition, we observed the characteristic Raman signal of H_2_O‐VII at high wavenumbers (≈3100 cm^−1^) confirming the presence of H_2_O in the sample chamber of the DAC (Figure S3). The Raman spectrum is in reasonable agreement with earlier studies at similar pressures,^[^ ^2^, ^29^ ^]^ but due to the proton disorder in the crystal structure a theoretical Raman spectrum of H_2_O‐VII could not be calculated. We used spatially resolved Raman spectroscopy to monitor the distribution of phases in the sample chamber of the DAC before the heating (Figure 2). These Raman maps unambiguously show that H_2_O‐VII crystals are present within a CO_2_‐I matrix.

At 9(1) GPa, the H_2_O + CO_2_ mixture was laser‐heated from both sides (Figure 2a). Laser‐heating was performed up to the beginning of optically detectable thermal radiation, which typically becomes visible at *T* ≈ 800 K.^[^ ^30^ ^]^ The heating time was ≈30 minutes. In this pressure range, the direct and indirect heating will not cause a phase transition in the starting materials H_2_O and CO_2_.^[^ ^25^, ^27^ ^]^ Consistent with this expectation, no difference was observed in the Raman spectra of CO_2_‐I and H_2_O‐VII before and after laser heating (Figure S3).

In order to understand if a reaction had occurred, we performed again spatially resolved Raman spectroscopy. We found, that in the laser heated regions the amount of CO_2_‐I and H_2_O‐VII is noticeably reduced, while a new unknown phase occurs in this region (Figure 2). The yet unknown phase shows a strong Raman mode at ≈1095 cm^−1^ (Figure 1d). Raman modes occurring at this wavenumber are characteristic for C–O stretching vibrations in a [CO_3_]^2−^‐group and are typically present in *sp* ^2^‐carbonates such as CaCO_3_.^[^ ^31^ ^]^ In addition three clearly identifiable new Raman modes appear in the region between 560 and 710 cm^−1^ (Figure 1e), which are also typical for compounds containing [CO_3_]^2−^‐groups. For these energies, the experimentally obtained Raman spectrum differs significantly from our calculated Raman spectra based on the reported H_2_CO_3_ phases *Pnma* and *P*2_1_/*c* at the same pressure (Figure 1b,c).^[^ ^17^, ^19^ ^]^

**Figure 3:**
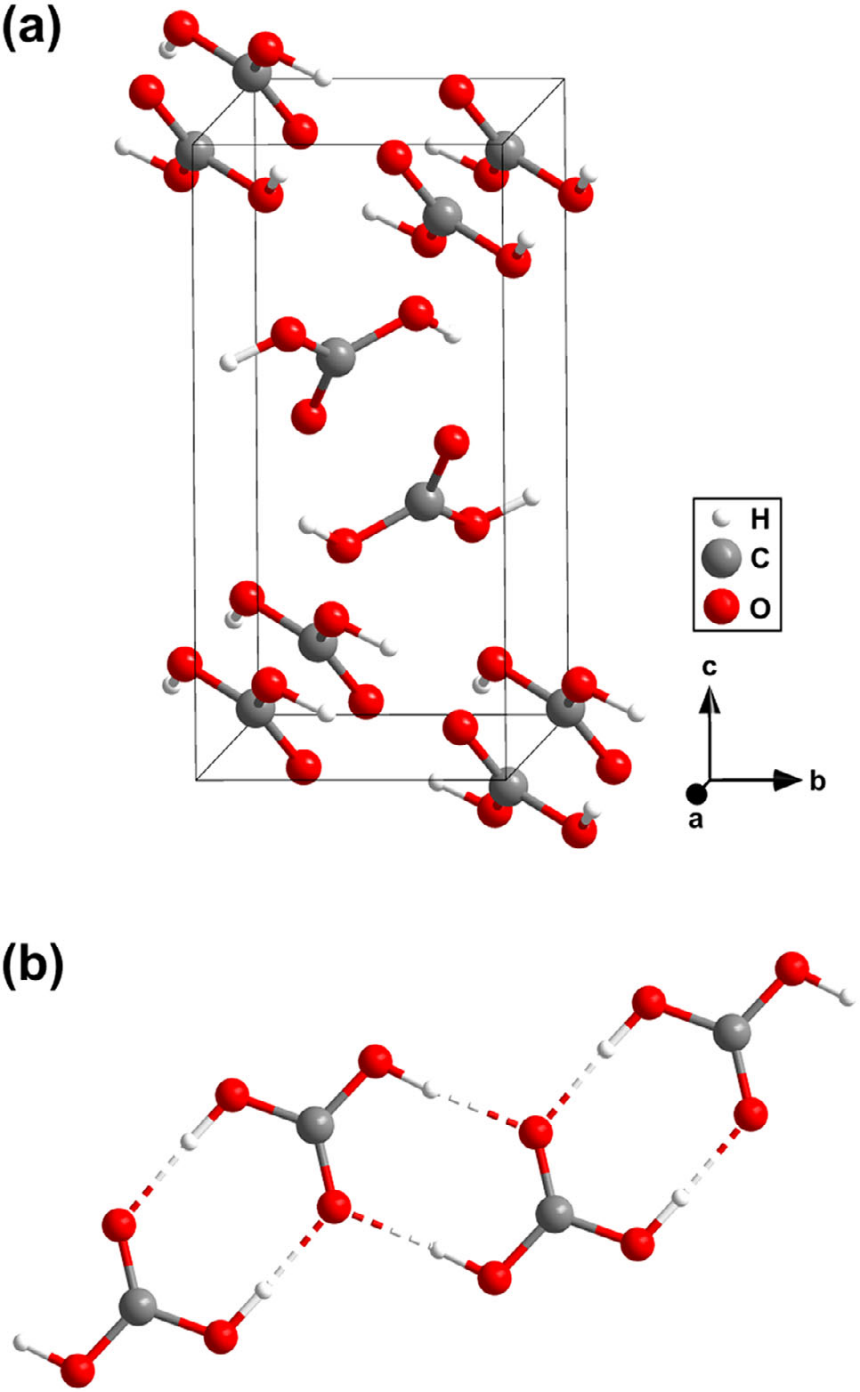
a) Monoclinic structure (*P*2_1_/*n*, *Z* = 4) of carbonic acid (H_2_CO_3_) at 8(1) GPa. b) Hydrogen bonds between the H_2_CO_3_ molecules in the crystal structure. The structural model is deposited at the CCDC.^[^ ^32^ ^]^

We then performed synchrotron X‐ray diffraction on a sample synthesized at 8(1) GPa. In the first step, we collected diffraction data on a grid across the sample chamber in order to locate promising positions for the subsequent collection of single‐crystal diffraction data. Afterward, we collected single‐crystal X‐ray diffraction data at selected locations using a ≈2 × 2 μ m^2^‐sized X‐ray beam (see Supporting Information). We solved the crystal structure of the unknown phase at 8(1) GPa. We found that it is a phase with H_2_CO_3_ composition and monoclinic space group *P*2_1_/*n* with *Z* = 4 (Figure 3a). The lattice parameters at 8(1) GPa are *a* = 4.428(1) Å, *b* = 4.498(1) Å, *c* = 9.034(4) Å, and β = 100.82(4)° (*V* = 176.7(1) Å^3^). The structure refinement ends with a *R*_1_‐value of 5.7%, which is indicative of a reliable structure refinement. The structure refinement is robust as it is based on a reflection‐to‐parameter‐ratio of 9.2:1 (see Supporting Information).

The crystal structure of H_2_CO_3_‐*P*2_1_/*n* is distinct from the structural models provided earlier.^[^ ^17^, ^19^ ^]^ We performed DFT‐based full geometry optimization on our structural model and the calculations accurately reproduce the experimentally determined structure (Table S1). Furthermore, the experimental Raman spectrum is in very good agreement with the one obtained from our calculations (Figure 1d), confirming that the structural model is appropriate. Raman modes between 300 and 1200 cm^−1^ are all related to H_2_CO_3_‐*P*2_1_/*n*. Specifically, the agreement between experiment and density functional perturbation theory (DFPT) calculations in the region between 550 and 700 cm^−1^ (Figure 1e) allows us to exclude the presence of the hypothetical polymorphs H_2_CO_3_‐*Pnma* and H_2_CO_3_‐*P*2_1_/*c*.^[^ ^17^, ^19^ ^]^ At low wavenumbers the strong Raman of CO_2_‐I overlaps with the Raman modes of H_2_CO_3_‐*P*2_1_/*n*. Nevertheless, we could identify several of the H_2_CO_3_‐*P*2_1_/*n* Raman modes at low wavenumbers <300 cm^−1^ (Figure S5). In addition, we used our structural model for DFPT calculations and calculated selected eigenvectors of the atomic displacements in the H_2_CO_3_ molecule. An example of the displacements in the H_2_CO_3_ molecule for the characteristic Raman mode at ≈620 cm^−1^ is shown in Figure 1f.

**Figure 4:**
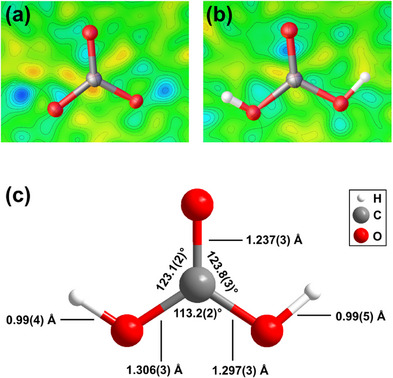
Difference Fourier map around the H_2_CO_3_ molecule at 8(1) GPa: a) refinement without hydrogen atoms and b) with hydrogen atoms connected to the oxygen atoms. c) Geometry of the H_2_CO_3_ molecule from single crystal structure refinement.

The crystal structure of H_2_CO_3_‐*P*2_1_/*n* is characterized by nearly planar H_2_CO_3_ molecules. Two H_2_CO_3_ molecules are connected by two hydrogen bonds with bond lengths of 1.71(6) Å/1.63(5) Å forming layers (Figure 3b). During the structure determination the hydrogen atoms could directly be located in the difference Fourier map (Figure 4a). After introducing the hydrogen atoms in the structural model, the residual electron density at these positions vanishes (Figure 4b). In addition, a refinement with hydrogen atoms causes a decrease of the *R*_1_‐value by ≈1%. With the synthesis and structure determination of Li[HC_2_O_5_], we demonstrated that we can reliably determine hydrogen positions with our experimental approach in light‐element containing compounds.^[^ ^33^ ^]^ We are aware, that the experimental error in the hydrogen position is larger than for the other atoms. Hence, we introduced a restraint for the O─H bond distance to the value derived from our DFT‐based calculations (≈1 Å), as it is generally accepted that DFT model calculations can reliably predict hydrogen positions.^[^ ^34^ ^]^ Otherwise, the experimental O─H bond would be slightly too short. No constrains or restrains were applied to the C–O–H angles or the atomic displacement parameters of hydrogen atoms.

The geometry of the H_2_CO_3_ molecule (Figure 4c) is in general agreement with the geometry of the H_2_CO_3_ molecule in hypothetical H_2_CO_3_‐*Pnma* and H_2_CO_3_‐*P*2_1_/*c*. However, in contrast to the results presented by Benz et al.,^[^ ^19^ ^]^ we observe two different C─O bond lengths.^[^ ^17^, ^19^ ^]^ The C─O bond to the isolated oxygen atom (1.237(3) Å) is significantly shorter than the other two C─O bonds, where the oxygen atom is connected to a hydrogen atom (1.306(3) Å/1.297(3) Å). This is reproduced by the DFT calculations. At 8 GPa the shorter C─O bond has a Mulliken bond population of 0.95 e^−^/Å^3^, while the longer ones have a bond population of 0.76 e^−^/Å^3^. The covalent O─H bond has a Mulliken bond population of 0.51 e^−^/Å^3^, while the O⋅⋅⋅H bond has a population of 0.15 e^−^/Å^3^. In contrast to the geometry of the H_2_CO_3_ molecules in H_2_CO_3_.H_2_O^[^ ^20^ ^]^ with *cis–trans* conformation, which was obtained in a similar pressure regime, the molecule in H_2_CO_3_‐*P*2_1_/*n* is in *cis–cis* conformation. In addition, the C─O bonds are noticeably longer in H_2_CO_3_.H_2_O (≈1.35–1.40 Å).^[^ ^20^ ^]^

We used the DFT calculations to derive the *p*, *V* relation for H_2_CO_3_‐*P*2_1_/*n*. The calculated *p*, *V* data were fitted with an equation of state to determine the bulk modulus (Figure S7). We obtained a bulk modulus of *K*_0_ = 14.2(4) GPa with *K*_p_ = 6.1(1). In addition, we used the DFT calculations to obtain phonon dispersion curves and phonon density of states (PDOS) for H_2_CO_3_‐*P*2_1_/*n*, H_2_CO_3_‐*Pnma* and H_2_CO_3_‐*P*2_1_/*c* (Figures S8 and S9). Our calculations of the PDOS show no negative frequencies and together with phonon dispersion curves they indicate that H_2_CO_3_‐*P*2_1_/*n* is dynamically stable at 8 GPa. Zero point energies and enthalpies are given in the Supporting Information.

Earlier calculations have already shown that there are several minima with very similar energies in the energy landscape describing the mutual orientation of H_2_CO_3_ in solids.^[^ ^17^, ^19^, ^35^ ^]^ This is in agreement with our calculations, where the three H_2_CO_3_ phases (*Pnma*, *P*2_1_/*c*, *P*2_1_/*n*) all have enthalpies within a few kJ/mol (see Supporting Information). The present study unambiguously connects diffraction experiments with spectroscopic data, and hence the presence of H_2_CO_3_‐*P*2_1_/*n* is now established, it is not unreasonable to expect that moderate changes in the *p*, *T*‐conditions may lead to the formation of one of the other polymorphs.

Laser‐heating a mixture of H_2_O + CO_2_ in further experiments between 5 and 13 GPa resulted in a Raman spectrum with the same characteristic Raman modes for H_2_CO_3_‐*P*2_1_/*n* as those observed at 9 GPa (Figure S4). In contrast, laser‐heating this mixture at pressures below or above this region (4(1) GPa or 15(1) GPa resulted in the occurrence of phases with different characteristic Raman modes, with currently unknown crystal structures. In addition, we observe that the Raman modes of H_2_O‐VII disappear in the laser‐heated region (Figure 2d). Hence, we speculate that a higher amount of water, present in other experiments performed at similar pressures, led to the formation of a hydrous phase in their experiments (H_2_CO_3_.H_2_O).^[^ ^2^, ^20^ ^]^ Moreover, we used noticeably higher temperatures during our synthesis than reported earlier.

## 3 Conclusion

In summary, we have synthesized H_2_CO_3_‐*P*2_1_/*n* by a reaction between H_2_O and CO_2_ at relatively low pressures and temperatures (5–13 GPa and ⩽800 K). For the first time, the crystal structure of a water‐free carbonic acid polymorph with H_2_CO_3_ composition was solved by synchrotron‐based single crystal X‐ray diffraction, without the need to introduce several constraints or restraints in the refinements. Our experimental structural data are in very good agreement with our DFT‐based full geometry optimizations. In addition, the reproduction of the experimental Raman data of H_2_CO_3_ by our DFPT calculations unambiguously confirms that the new phase is H_2_CO_3_‐*P*2_1_/*n*. The crystal structure described here is distinct from the structural models derived earlier at other *p*, *T*‐conditions. It is therefore now of great interest to understand the stability field of H_2_CO_3_‐*P*2_1_/*n*, in order to determine whether additional polymorphs exist.

## Conflict of Interest

The authors declare no conflict of interest.

## Supporting information

Supporting Information

## Data Availability

All study data are included in the article and/or in the SI. Structural models had been deposited at the CCDC.^[^ ^32^ ^]^
