# Supplementary material for: Crystal Structure of Carbonic Acid (H2CO3) at Elevated Pressures from Single‐Crystal Diffraction
Source: Chemistry. 2025 Jul 9;31(50):e202501964. doi: 10.1002/chem.202501964 (PMC12415326; doi:10.1002/chem.202501964)
Supplement: Supplementary file 1 — Supporting Information [file CHEM-31-e202501964-s001.pdf]

Supplementary material: Crystal Structure of Carbonic Acid (H_2CO_3) at Elevated Pressures from Single-Crystal Diffraction

Dominik Spahr*[,a], Elena Bykova[a], Lkhamsuren Bayarjargal[a], Maxim Bykov[b], Lukas Brüning[b], Valentin Kovalev[a], Victor Milman[c], Nico Giordano[d], Hanns-Peter Liermann[d], Björn Winkler[a]

[a]*Goethe University Frankfurt, Institute of Geosciences, Altenhöferallee 1, 60438 Frankfurt, Germany*
[b]*Goethe University Frankfurt, Institute of Inorganic and Analytical Chemistry, Max-von-Laue-Straße 7, 60438 Frankfurt, Germany*
[c]*Dassault Systèmes BIOVIA, 334 Cambridge Science Park, Cambridge CB4 0WN, United Kingdom*
[d]*Deutsches Elektronen-Synchrotron DESY, Notkestrasse 85, 22607 Hamburg, Germany*

1. Methods

1.1. *High-pressure experiments*

The high-pressure experiments were carried out in Boehler-Almax type DACs.[1] We employed DACs equipped with diamonds having an opening angle of 70° and with 350 µm culet size on both sides. We used Re-gaskets which we pre-indented to thicknesses of ≈ 45 µm. Afterwards, we drilled gasket holes with 60–120 µm diameter using a custom-built laser set-up. In order to determine the pressure during the experiment, we added a ruby chip on the culet of the bottom diamond, prior to the cryogenic loading of the DAC. The pressure was determined by measuring the shift of the ruby fluorescence and we assume an error of 6% due to non-hydrostatic conditions.[2]

For the cryogenic loading the DAC was placed on a liquid nitrogen cooled Cu-holder and cooled down using a custom-built cryogenic loading system (see Spahr *et al.*[3]). In the first step, the DAC was cooled down in air without any purge gas and we monitored the precipitation of H_2O in the sample chamber using an optical microscope and a camera. After a sufficient amount of H_2O-ice was condensed into the sample chamber, we switched on the CO_2 gas jet, which was directly directed at the gap between upper diamond and the gasket. Finally, the DAC was cooled down further to ≈ 100 K and CO_2-I (dry ice) was directly condensed into the gasket hole from the CO_2 gas jet. We used a small nozzle to align the CO_2 gas jet with 5 l min^{-1}. For the gas-jet during the cryogenic loading we used CO_2 gas as purchased (Nippon gases, purity ≥ 99.996%). After the sample chamber was completely covered with dry-ice, the DAC was tightly closed again and compressed to the target pressure without intermediate heating.

1.2. *Laser heating*

At the target pressures of the experiment the H_2O + CO_2 mixtures were laser-heated from both sides using a custom-built set-up equipped with a Coherent Diamond K-250 pulsed CO_2 laser ($\lambda = 10600$ nm).[4] The laser power was adjusted to achieve a coupling of the laser to the sample using a laser power of ≤ 1 W. The samples were heated until beginning of thermal radiation in the visible spectrum was visible, which is typically at $T \approx 800$ K.[5] We assume that the highest temperature achieved during the laser heating was $T_{\max} \approx 800(200)$ K. The temperatures were determined by the two-color pyrometer method, employing Planck and Wien fits.[6] The heating time was ≈ 30 min. It is well established that laser-heating in DACs always suffers from large temperature gradients and the actual temperature is strongly dependent on the coupling of the laser with the sample, especially at lower temperatures. We estimate an uncertainty of at least ±20% of the nominal temperature in the laser-heated region depending on the focus of the laser beam, based on typical 2D temperature-gradient determination experiments performed in DACs.[7]

1.3. *Raman spectroscopy*

Raman spectroscopy was performed in DACs using an Oxford Instruments WITec alpha 300R Raman imaging microscope. The Raman microscope was equipped with an Olympus SLMPan N 50× objective. The measurements were performed using the 532 nm laser. We employed the 1800 grooves mm^{-1} grating of the WITec UHTS 300S (VIS-NIR) spectrograph in combination with an Andor DR316B-LDC-DD CCD detector for the measurements. The applied laser power was 100 mW on the sample and the spot size of the Raman laser was ≈ 0.8 µm. We assume a depth resolution of ≈ 6 µm in the direction of the laser beam. Raman maps were measured on a grid with a step-size of 1 µm. The background of the Raman spectra was corrected using the software package Fityk.[8]

1.4. *Single-crystal synchrotron X-ray diffraction*

Single-crystal synchrotron X-ray diffraction was carried out at the synchrotron PETRA III (DESY) in Hamburg, Germany, at the extreme conditions beamline P02.2.[9] The beam size on the sample was ≈ 2 × 2 µm^2 (FWHM), focused by Kirkpatrick Baez mirrors. The diffraction data were collected using a Perkin Elmer XRD1621 detector, a wavelength of 0.2910 Å (42.7 keV) and a sample to detector distance of 405.7 mm. We rotated the DAC by ±33° around the vertical axis perpendicular to the beam while collecting frames in 0.25° steps with 4 s acquisition time per frame.

The sample to detector distance was calibrated using the powder diffraction pattern of a CeO_2 standard in conjunction with the software DIOPTAS.[10] The diffractometer/detector geometry for the analysis of the single crystal diffraction data was calibrated using diffraction

*d.spahr@kristall.uni-frankfurt.de

data collected from a single crystal of enstatite ($MgSiO_3$) in a DAC at ambient pressure. After the data collection, the reflections were indexed and integrated employing CrysAlis^{PRO} (version 43.67a).[11] We used the Domain Auto Finder program (DAFi) to find possible single-crystal domains for the subsequent data reduction.[12] The structure solution and refinement were performed using the software package OLEX2 employing SHELXT for the crystal structure determination and SHELXL for the refinement.[13,14,15]

1.5. Density functional theory-based calculations

First-principles calculations were carried out within the framework of density functional theory (DFT), employing the Perdew-Burke-Ernzerhof (PBE) exchange-correlation functional and the plane wave/pseudopotential approach implemented in the CASTEP simulation package.[16,17,18] "On the fly" norm-conserving or ultrasoft pseudopotentials generated using the descriptors in the CASTEP data base were employed in conjunction with plane waves up to a kinetic energy cutoff of 1020 eV or 630 eV, for norm-conserving and ultrasoft pseudopotentials, respectively. The accuracy of the pseudopotentials is well established.[19] A correction scheme for van der Waals (v.d.W.) interactions was applied in the DFT-calculations. We employed the correction scheme developed by Tkatchenko and Scheffler.[20] A Monkhorst-Pack grid was used for Brillouin zone integrations.[21] We used a distance between grid points of <0.023 Å^{-1}. Convergence criteria for geometry optimization included an energy change of $<5 \times 10^{-6}$ eV atom^{-1} between steps, a maximal force of <0.008 eV Å^{-1} and a maximal component of the stress tensor <0.02 GPa. Phonon frequencies were obtained from density functional perturbation theory (DFPT) calculations.[22,23] Raman intensities were computed using DFPT with the "$2n+1$" theorem approach.[24]

2. Results

2.1. *Single-crystal synchrotron X-ray diffraction*

We employed synchrotron X-ray diffraction in order to determine the crystal structure of the unknown phase after laser-heating at 8(1) GPa. First, we collected X-ray diffraction data on a grid across the gasket hole using a spot size of $\approx 2 \times 2$ µm^2. Afterwards, we collected diffraction data suitable for single-crystal X-ray diffraction analysis on selected locations, where unknown reflections occur in the X-ray diffraction map, which cannot be assigned to known phases. Fig. S 1 a shows reciprocal space reconstruction for ($hk\bar{1}$) plane (*unwarped*-image) after data processing in CrysAlis on the location selected for the single-crystal structure solution. Besides the reflection of the unknown phase, we observed reflections and powder rings of CO_2-I, H_2O-VII and of the diamonds in the diffraction data.

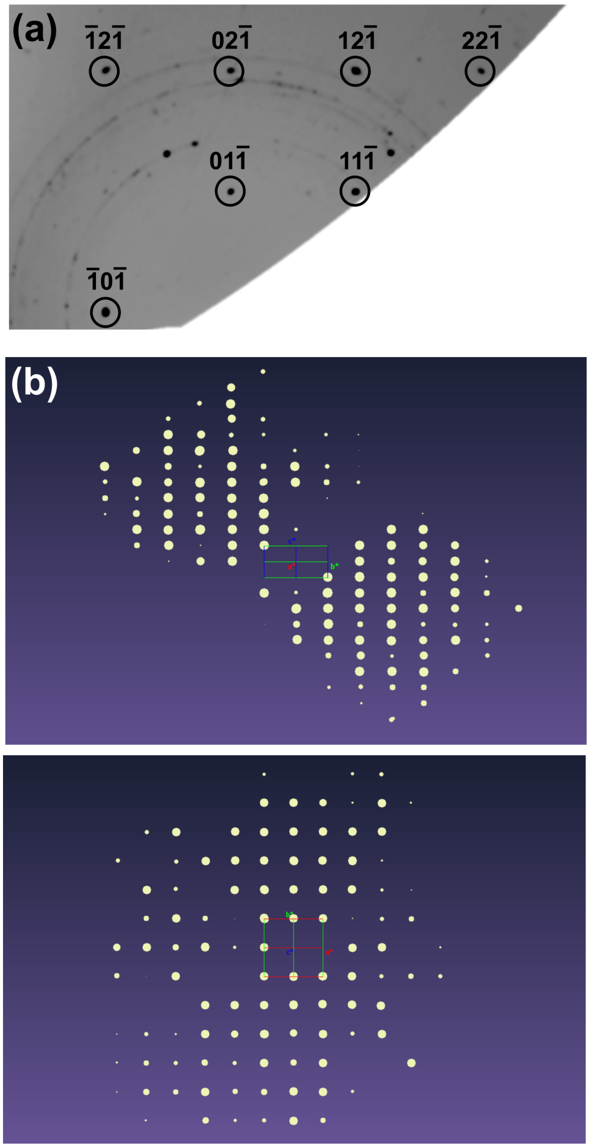

Figure S 1: (a) Reciprocal space reconstruction for ($hk\bar{1}$) plane. (b) Schematic depiction of the reflections in reciprocal space using the Ewald-Explorer in CrysAlis after data reduction which were later used for the refinement. Projections of the reciprocal space are shown along a^* (top) and c^* (bottom).

Fig. S 1 b shows projections of the reciprocal space along a^* and c^*. The effect of the shading of diffracted beams due to the metallic body of the DAC can be noticed. Nevertheless, the coverage of the reciprocal space is good for a DAC experiment.

Table S 1: Structural parameters of H_2CO_3-$P2_1/n$ at 8(1) GPa from synchrotron single-crystal structure solution (ambient temperature) in comparison to data derived from DFT calculations at the same pressure (athermal limit).

	**Single Crystal**	**DFT**
**Crystal data**		
Crystal system	monoclinic	monoclinic
Space group	$P2_1/n$	$P2_1/n$
Chemical formula	H_2CO_3	H_2CO_3
M_r	62.03	62.03
a (Å)	4.4278(13)	4.4511
b (Å)	4.4978(9)	4.4717
c (Å)	9.034(4)	9.1669
α (°)	90.0	90.0
β (°)	100.82(4)	101.90
γ (°)	90.0	90.0
V (Å^3)	176.71(10)	178.54
Z	4	4
**Data collection**		-
F_{000}	128	-
θ range (°)	2.08–13.99	-
measured reflections	690	-
independent reflections	416	-
reflections $I > 2\sigma(I)$	290	-
R_{int}	0.021	-
		-
**Refinement**		-
$R_1[I > 2\sigma(I)]$, $wR_2(I)$	0.057, 0.140	-
No. of reflections	416	-
No. of parameters	45	-
No. of restraints	2	-
No. of constraints	0	-
$\Delta\rho_{max}$, $\Delta\rho_{min}$ (e Å^{-3})	0.34, −0.29	-

We solved the crystal structure of the unknown phase in space group $P2_1/n$ (No. 14) with $Z = 4$ and with a chemical composition of H_2CO_3. The R_1-value (5.7%) of the refinement is reasonable for a DAC experiment, while the reflection to parameter ratio (9.2:1) is satisfactory. The crystallographic parameters of H_2CO_3-$P2_1/n$ at 8(1) GPa are listed in Table S 1 and in comparison to data derived from the DFT calculations. The displacement parameters of the carbon and the oxygen atoms were refined anisotropically. The displacement parameters of the hydrogen atoms were refined isotropically. All displacement parameters were refined without constraints or restraints. We introduced a distance restraint in order to ensure that the O–H bond length is in agreement with the value derived from the DFT calculations. It should be noted that the $F^2/\sigma(F^2)$-value is very high (≈ 130 up to 0.8 Å^{-1} and ≈ 58 up to 0.5 Å^{-1}) even if only light elements (H, C, O) are present in the crystal structure and the diffrac-

tion experiment was performed on a multi-grain reaction product inside a DAC at high pressure.

Figure S 2 shows the Raman-map as well as the XRD-maps for H_2CO_3-$P2_1/n$ after the experiment where we solved the crystal structure from synchrotron single-crystal structure solution at 8(1) GPa. The peaks recognized in the XRD-map at $2\theta \approx 4.16°$ (Fig. 2 c) and $2\theta \approx 4.58°$ (Fig. 2 d) correspond to $(0\bar{1}0)$ and (101) lattice planes, respectively.

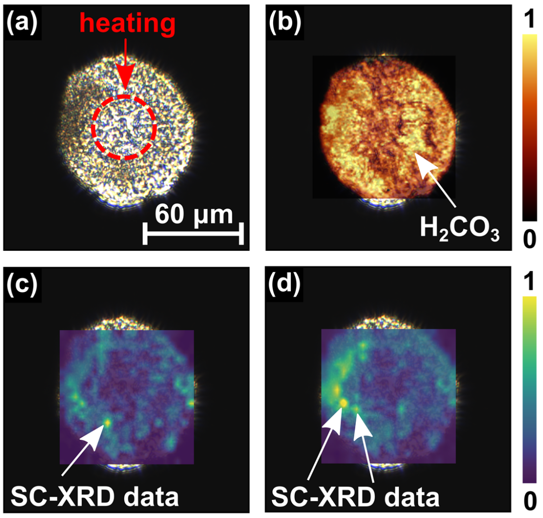

Figure S 2: a) Gasket hole of the DAC with the H_2O + CO_2 mixture after laser heating at 8(1) GPa up to temperatures below $\leq 800(200)$ K. (b) An overlay showing the distribution of H_2CO_3 according to the 2D-Raman map. XRD-maps as an overlay over the photograph of the gasket hole. The color on the overlay is proportional to the intensity of the following reflections: (c) $2\theta \approx 4.16°$ and (d) $2\theta \approx 4.58°$. Arrows point on the positions where SC-XRD was collected.

2.2. H_2O content in the sample chamber

We used Raman spectroscopy in oder to understand the H_2O content in the sample chamber of the DAC before and after the synthesis of H_2CO_3-$P2_1/n$ (Fig. S 3).

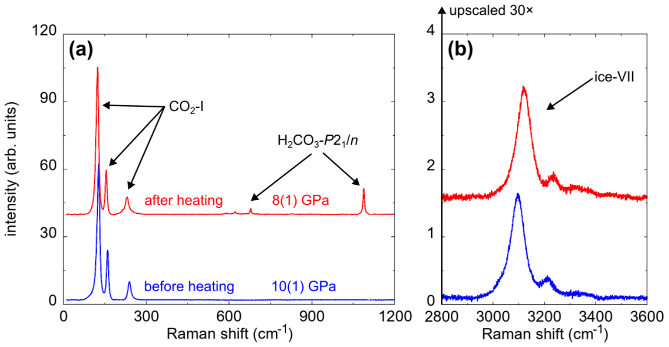

Figure S 3: Raman spectra of the H_2O + CO_2 mixture before and after the laser heating at 10(1) GPa measured. After the heating the pressures drops to $\approx$ 8 GPa.

In addition, to the strong characteristic Raman modes of CO_2-I at low wavenumber, we observed a Raman signal of H_2O-VII at high wavenumbers ($\approx 3100\ \mathrm{cm}^{-1}$) before the heating in agreement with earlier studies.[25,26] The intensity of the CO_2-I Raman modes is approximately 30 times largen than of H_2O-VII. We did not observe a difference in the Raman spectra of CO_2-I and H_2O-VII measured at the same position in the DAC before and after laser heating (Fig. S 3). Only a slight shift of the peak positions is present due to the pressure drop during the laser heating. In addition, the characteristic Raman modes of H_2CO_3-$P2_1/n$ are present after the heating.

2.3. Synthesis of H_2CO_3 at different pressures

H_2CO_3-$P2_1/n$ was synthesized by laser-heating mixtures of H_2O + CO_2 in a different experiments between 5–13 GPa. The experimental Raman data between 5–13 GPa show the same characteristic Raman modes of H_2CO_3-$P2_1/n$ (Fig. S 4). At lower as well as at higher pressures unknown phases are formed, which significantly differ in their Raman spectra (Fig. S 4).

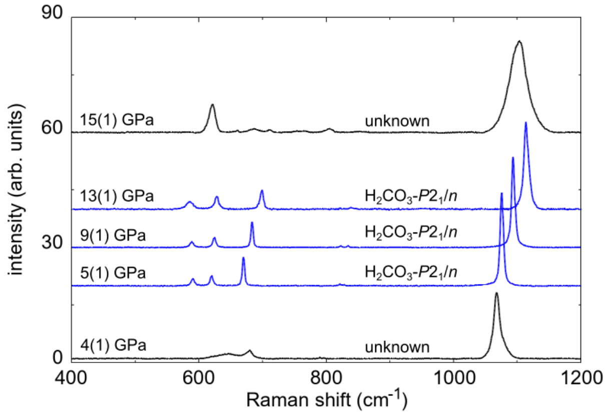

Figure S 4: Raman spectra after laser-heating mixtures of H_2O + CO_2 in different experiments between 4–15 GPa.

The very strong Raman modes of CO_2-I overlap with the Raman modes of H_2CO_3-$P21/n$ at low wavenumbers. Nevertheless, we could identify several of the H_2CO_3-$P21/n$ Raman modes at wavenumbers $< 300\ \mathrm{cm}^{-1}$, which are in good agreement with our DFT-based calculations.

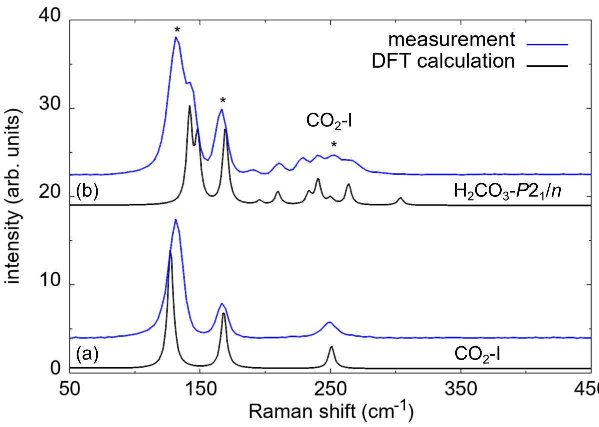

Figure S 5: Raman spectra for (a) CO_2-I and (b) H_2CO_3-$P2_1/n$. Experimental Raman spectra are shown in blue and DFT-based calculations (rescaled by 1–3 %) are shown in black. Peaks of CO_2-I are marked by an asterisk ($*$).

2.4. DFT calculations on H_2CO_3

DFT-calculation were employed to provide theoretical Raman spectra of the three H_2CO_3 phases ($Pnma$, $P2_1/c$, $P2_1/n$) discussed in the manuscript (Fig. S 6).

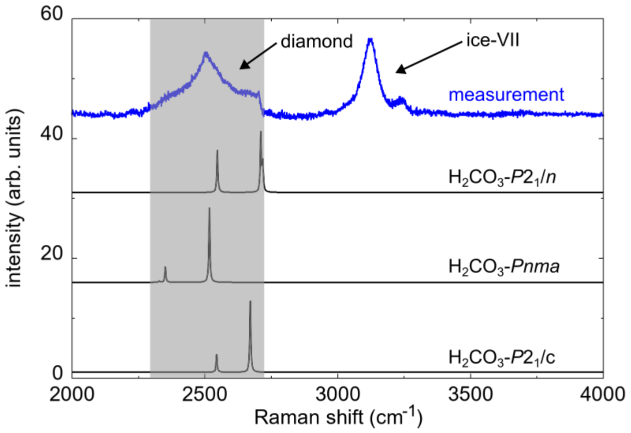

Figure S 6: Calculated Raman spectra for H_2CO_3-$P2_1/n$, H_2CO_3-$Pnma$[27] and H_2CO_3-$P2_1/c$[28] in comparison the experimental data at high wavenumber.

From the DFT-calculations we found that all three H_2CO_3 phases will not have characteristic Raman modes in the region of H_2O-VII at high wavenumbers ($\approx$ 2800–3600 cm^{-1}). The characteristic Raman modes of the three H_2CO_3 phases ($Pnma$, $P2_1/c$, $P2_1/n$) discussed in our study may not be observed in a DAC due to the overtone of the stressed diamond in the broad region around 2500 cm^{-1} (Fig. S 6).

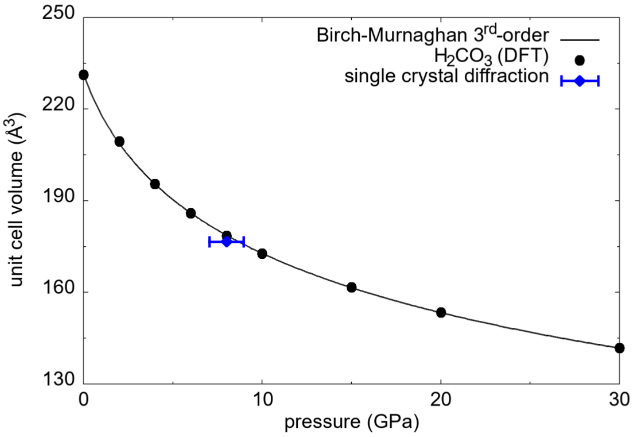

Figure S 7: A Birch-Murnaghan EoS was fitted to the theoretical p, V-data of H_2CO_3-$P2_1/n$ obtained by DFT-based calculations between 0–30 GPa. The experimentally obtained unit cell volume from the single crystal structure solution at 8(1) GPa is shown for comparison.

From the DFT-based calculations we derived the pressure depending unit cell volume for H_2CO_3-$P2_1/n$ in the pressure range between 0 GPa and 30 GPa. The p, V data from the calculations were fitted with a 3rd-order Birch-Murnaghan equation of states (EoS) using the software package EOSFit7-GUI in order to determine the bulk modulus K_0 (Fig. S 7).[29,30,31] The theoretical bulk modulus of H_2CO_3-$P2_1/n$ and its derivative in the range between 0–30 GPa derived from the p, V relation is K_0 = 14.2(4) GPa with K_p = 4.34(1). The experimentally determined unit cell volume at 8(1) GPa is in very good agreement with the theoretical data derived from our calculations (Fig. S 7).

We employed the DFT calculations to derive the phonon dispersion curves for H_2CO_3-$P2_1/n$, H_2CO_3-$P2_1/c$ and H_2CO_3-$Pnma$ (Fig. S 8). The phonon dispersion curves for H_2CO_3-$P2_1/n$ show that the compound is dynamically stable at 8 GPa. However, as we believe that the intrinsic inaccuracies of the approach employed here (van der Waals corrected DFT-GGA-PBE approach in the athermal limit and the harmonic approxi-mation) would severely limit the accuracy of computed thermodynamic properties, the latter were not computed.

The zero-point energies for the three compounds are: 4.427 eV per unit cell (H_2CO_3-$P2_1/n$), 4.418 eV per unit cell (H_2CO_3-$P2_1/c$) and 4.404 eV per unit cell (H_2CO_3-$Pnma$). The computed enthalpies for the three compounds are: 144838 kJ/mol (H_2CO_3-$P2_1/n$), 144828 kJ/mol (H_2CO_3-$P2_1/c$) and 144837 kJ/mol (H_2CO_3-$Pnma$).

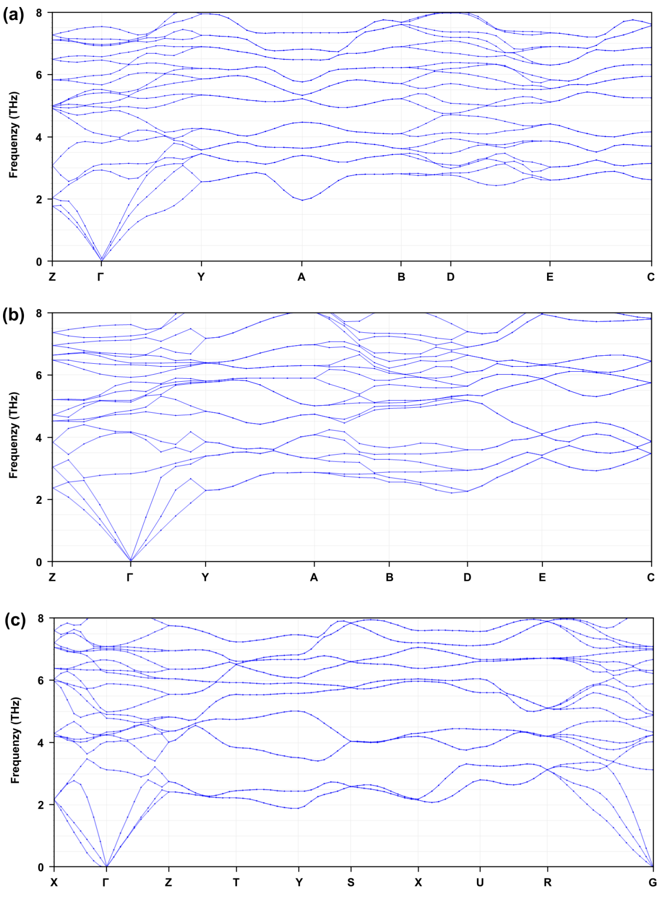

Figure S 8: Phonon dispersion curves for (a) H_2CO_3-$P2_1/n$, (b) H_2CO_3-$P2_1/c$[28] and (c) H_2CO_3-$Pnma$[27] at 8 GPa from DFT-based calculations.

In addition, we used our DFT-based calculations to obtain the phonon density of states for the three H_2CO_3 phases ($Pnma$, $P2_1/c$, $P2_1/n$) discussed in our study (Fig. S 9). The calculations show no negative frequencies for H_2CO_3-$P2_1/n$ and reveal that this compound is dynamically stable at elevated pressures in the harmonic approximation and the athermal limit.

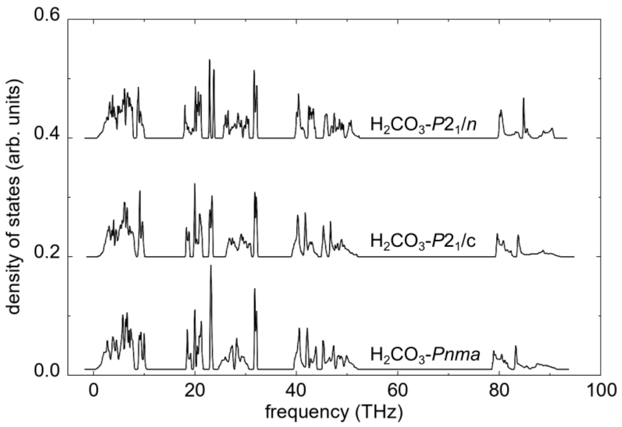

Figure S 9: Phonon density of states for (a) H_2CO_3-$P2_1/n$, (b) H_2CO_3-$P2_1/c$[28] and (c) H_2CO_3-$Pnma$[27] at 8 GPa from DFT-based calculations.

References

(1) Boehler, R. New diamond cell for single–crystal X-ray diffraction. *Rev. Sci. Instrum.* **2006**, *77*, 115103–1–115103–3, DOI: 10.1029/JB091iB05p04673

(2) Mao, H. K.; Xu, J.; Bell, P. M. Calibration of the ruby pressure gauge to 800 kbar under quasi-hydrostatic conditions. *J. Geophys. Res.* **1986**, *91*, 4673–4676, DOI: 10.1029/JB091iB05p04673

(3) Spahr, D.; König, J.; Bayarjargal, L.; Luchitskaia, R.; Milman, V.; Perlov, A.; Liermann, H.-P.; Winkler, B.; Synthesis and Structure of Pb[C_2O_5]: An Inorganic Pyrocarbonate Salt. *Inorg. Chem.* **2022**, *61*, 9855–9859, DOI: 10.1021/acs.inorgchem.2c01507

(4) Scelta, D.; Ceppatelli, M.; Ballerini, R.; Hajeb, A.; Peruzzini, M.; Bini, R. Sprayloading: A cryogenic deposition method for diamond anvil cell. *Rev. Sci. Instrum.* **2018**, *89*, 053903, DOI: 10.1063/1.5011286

(5) Bayarjargal, L.; Fruhner, C.-J.; Schrodt, N.; Winkler, B. $CaCO_3$ phase diagram studied with Raman spectroscopy at pressures up to 50 GPa and high temperatures and DFT modeling. *Phys. Earth Planet. Inter.* **2018**, *281*, 31–45, DOI: 10.1016/j.pepi.2018.05.002

(6) Draper, J. W. On the production of light by heat. *Lond. Edinb. Dublin Philos. Mag. J. Sci.* **1847**, *30*, 345–360, DOI: 10.1080/14786444708647190

(7) Benedetti, L. R.; Loubeyre, P. Temperature gradients, wavelength-dependent emissivity, and accuracy of high and very-high temperatures measured in the laser-heated diamond cell. *High Press. Res.* **2004**, *24*, 423–455, DOI: 10.1080/08957950412331331718

(8) Du, Z.; Amulele, G.; Benedetti, L. R.; Lee, K. K. M. Mapping temperatures and temperature gradients during flash heating in a diamond-anvil cell. *Rev. Sci. Instrum.* **2013**, *84*, 075111, DOI: 10.1063/1.4813704

(9) Wojdyr, M. *Fityk*: a general-purpose peak fitting program. *J. Appl. Cryst.* **2010**, *43*, 1126–1128, DOI: 10.1107/S0021889810030499

(10) Liermann, H.-P.; Konôpková, Z.; Morgenroth, W.; Glazyrin, K.; Bednarčik, J.; McBride, E. E.; Petitgirard, S.; Delitz, J. T.; Wendt, M.; Bican, Y.; Ehnes, A.; Schwark, I.; Rothkirch, A.; Tischer, M.; Heuer, J.; Schulte-Schrepping, H.; Kracht, T.; Franz, H. The Extreme Conditions Beamline P02.2 and the Extreme Conditions Science Infrastructure at PETRAIII. *J. Synchrotron Radiat.* **2014**, *22*, 908–924, DOI: 10.1107/S1600577515005937

(11) Prescher, C.; Prakapenka, V. B. *DIOPTAS*: a program for reduction of two-dimensional X-ray diffraction data and data exploration. *High. Press. Res.* **2015**, *35*, 223–230, DOI: 10.1080/08957959.2015.1059835

(12) Agilent, CrysAlis PRO, Yarnton, England, **2014**

(13) Aslandukov, A.; Aslandukov, M.; Dubrovinskaia, N.; Dubrovinsky, L. *Domain Auto Finder* (*DAFi*) program: the analysis of single-crystal X-ray diffraction data from polycrystalline sample. *J. Appl. Cryst.* **2022**, *55*, 1383–1391, DOI: 10.1107/S1600576722008081

(14) Dolomanov, O. V.; Bourhis, L. J.; Gildea, R. J.; Howard, J. A. K.; Puschmann, H. *OLEX2*: a complete structure solution, refinement and analysis program. *J. Appl. Cryst.* **2009**, *42*, 339–341, DOI: 10.1107/S0021889808042726

(15) Sheldrick, G. M. *SHELXT* — Integrated space-group and crystal-structure determination. *Acta. Cryst.* **2015**, *A71*, 3–8, DOI: 10.1107/S2053273314026370

(16) Sheldrick, G. M. Crystal structure refinement with *SHELXL*. *Acta. Cryst.* **2015**, *C71*, 3–8, DOI: 10.1107/S2053229614024218

(17) Hohenberg, P.; Kohn, W. Inhomogeneous Electron Gas. *Phys. Rev.* **1967**, *136*, B864–B871, DOI: 10.1103/PhysRev.136.B864

(18) Perdew, J. P.; Burke, K.; Ernzerhof, M. Generalized Gradient Approximation Made Simple. *Phys. Rev. Lett.* **1996**, *77*, 3865–3868, DOI: 10.1103/PhysRevLett.77.3865

(19) Clark, S. J.; Segall, M. D.; Pickard, C. J.; Hasnip, P. J.; Probert, M. I. J.; Refson, K.; Payne, M. C. First principles methods using CASTEP. *Z. Kristallogr.* **2005**, *220*, 567–570, DOI: 10.1524/zkri.220.5.567.65075

(20) Lejaeghere, K.; Bihlmayer, G.; Björkman, T.; Blaha, P.; Blügel, S.; Blum, V.; Caliste, D.; Castelli, I. E.; Clark, S. J.; Dal Corso, A. et al. Reproducibility in density functional theory calculations of solids. *Science* **2016**, *351*, aad3000, DOI: 10.1126/science.aad3000

(21) Tkatchenko, A.; Scheffler, M. Accurate Molecular Van Der Waals Interactions from Ground-State Electron Density and Free-Atom Reference Data. *Phys. Rev. Lett.* **2009**, *102*, 073005, DOI: 10.1103/PhysRevLett.102.073005

(22) Monkhorst, H. J.; Pack, J. D. Special points for Brillouin-zone integrations. *Phys. Rev. B* **1976**, *13*, 5188–5192, DOI: 10.1103/PhysRevB.13.5188

(23) Baroni, S.; de Gironcoli, S.; Dal Corso, A.; Giannozzi, P. Phonons and related crystal properties from density-functional perturbation theory. *Rev. Mod. Phys.* **2001**, *73*, 515–562, DOI: 10.1103/RevModPhys.73.515

(24) Refson, K.; Tulip, P. R.; Clark, S. J. Variational density-functional perturbation theory for dielectrics and lattice dynamics. *Phys. Rev. B* **2006**, *73*, 155114, DOI: 10.1103/PhysRevB.73.155114

(25) Miwa, K. Prediction of Raman spectra with ultrasoft pseudopotentials. *Phys. Rev. B* **2011**, *84*, 094304, DOI: 10.1103/PhysRevB.84.094304

(26) Hsieh, W.-P.; Chien, Y.-H. High pressure Raman spectroscopy of H_2O-CH_3OH mixtures. *Sci. Rep.* **2015**, *5*, 8532, DOI: 10.1038/srep08532

(27) Berni, S.; Scelta, D.; Romi, S.; Fanetti, S.; Alabarse, F.; Pagliai, M.; Bini, R. Exploring High-Pressure Polymorphism in Carbonic Acid through Direct Synthesis from Carbon Dioxide Clathrate Hydrate. *Angew. Chem. Int. Ed.* **2024**, *63*, e202403953, DOI: 10.1002/anie.202403953

(28) Saleh, G.; Oganov, A. R. Novel Stable Compounds in the C-H-O Ternary System at High Pressure. *Sci. Rep.* **2016**, *6*, 32486, DOI: 10.1038/srep32486

(29) Benz, S.; Chen, D.; Möller, A.; Hofmann, M.; Schnieders, D.; Dronskowski, R. The Crystal Structure of Carbonic Acid. *Inorganics* **2022**, *10*, 132, DOI: 10.3390/inorganics10090132

(30) Murnaghan, F. The Compressibility of Media under Extreme Pressures. *Proc. Natl. Acad. Sci.* **1944**, *30*, 244–247, DOI: 10.1073/pnas.30.9.244

(31) Birch, F. Finite Elastic Strain of Cubic Crystals. *Phys. Rev.* **1947**, *71*, 809–824, DOI: 10.1103/PhysRev.71.809

(32) Gonzalez-Platas, J.; Alvaro, M.; Nestola, F.; Angel, R. *EosFit7-GUI*: a new graphical user interface for equation of state calculations, analyses and teaching. *J. Appl. Cryst.* **2016**, *49*, 1377–1382, DOI: 10.1107/S1600576716008050
